# Perioperative Anaesthetic Management of Penetrating Anterior Neck Injuries With Laryngotracheal Disruption: A Case Series

**DOI:** 10.7759/cureus.117652

**Published:** 2026-10-08

**Authors:** Deepali Chandra, Farhan Z Sheikh, Parul Saxena, Rati Prabha

**Affiliations:** 1 Department of Anesthesiology, King George's Medical University, Lucknow, IND

**Keywords:** airway management, anaesthesia, neck injuries, tracheal injuries, trauma

## Abstract

Penetrating anterior neck injuries with laryngotracheal disruption create an immediate airway challenge because instrumentation may worsen tissue disruption or air leakage. We describe the perioperative anaesthetic management of three patients requiring emergency airway control, exploration and definitive repair. The injuries differed in mechanism, anatomical extent, associated cervical injury and physiological status. In the first patient, video laryngoscopy with manual in-line stabilisation was planned because of an associated C3 vertebral fracture; in the other two, direct laryngoscopy allowed careful advancement of a smaller tracheal tube beyond the injured segment. Definitive tracheostomy and repair were subsequently performed in all three patients. Two patients required postoperative mechanical ventilation for approximately 12 and 18 hours, respectively. All three patients had satisfactory airway and functional outcomes. These cases emphasise anatomy-driven airway planning, controlled distal airway access, immediate readiness for surgical airway rescue and close coordination between anaesthesia and otolaryngologist (ENT) teams.

## Introduction

Penetrating neck trauma is uncommon but can involve the airway, larynx, pharynx, oesophagus and major vascular structures, with potentially rapid deterioration [1]. The clinical presentation can vary considerably depending on the structures involved and the extent of tissue disruption. Patients may present with airway compromise, respiratory distress, bleeding, subcutaneous emphysema or physiological instability, and the initial clinical condition may change rapidly. Contemporary management emphasises physiological status, clinical signs and selective investigation rather than relying solely on the anatomical zone of the external wound [1,2].

Airway management remains one of the most critical aspects of care in patients with penetrating neck injuries involving the laryngotracheal framework. Disruption of the normal airway anatomy can result in loss of airway continuity, air leakage and distortion of the tracheal or laryngeal lumen. When the laryngotracheal framework is disrupted, airway instrumentation may create a false passage or worsen the injury [3,4]. The presence of an open airway injury also makes maintenance of effective ventilation and oxygenation particularly challenging, while manipulation of the injured airway may result in further tissue disruption.

The anaesthetic management of these patients therefore requires careful assessment of the level and extent of airway injury, associated cervical or laryngeal injuries and the patient's physiological status. The airway strategy may need to be modified according to the individual anatomical findings, with careful airway instrumentation and readiness for immediate surgical airway access. Previous reports have described airway management strategies in penetrating neck trauma, including careful advancement of the tracheal tube beyond the injured segment under direct visualization [5]. Reports of severe cut-throat injuries have also highlighted the challenges of maintaining and securing the airway in the presence of major airway disruption [6-8]. Coordination between the anaesthesia and surgical teams is particularly important because definitive airway control and surgical repair may need to occur simultaneously or in rapid succession.

We describe three patients with penetrating anterior neck injuries requiring emergency airway control, exploration and definitive repair. The purpose of presenting these cases is to highlight the practical challenges of airway management in laryngotracheal disruption and to demonstrate how the airway approach was individualised according to the anatomical extent of injury, associated injuries and physiological status of each patient.

## Case presentation

Case 1

An 18-year-old man sustained a cut-throat injury after falling from a motorcycle onto roadside fencing wire. A 12 × 3.5 cm horizontal laceration over the thyroid cartilage was associated with a C3 vertebral body fracture (Figure 1). Air escaped through the wound; pulse was 100/min, blood pressure 98/62 mmHg and room-air SpO₂ 85%. External pressure, oxygen, intravenous access and crystalloid resuscitation were instituted before theatre transfer. Because cervical movement was restricted, video laryngoscopy with manual in-line stabilisation was performed, with a surgical airway immediately available. After preoxygenation, anaesthesia was induced with propofol and vecuronium. A 7.5-mm tracheal tube was passed beyond the injured segment and secured at 21 cm.

**Figure 1:**
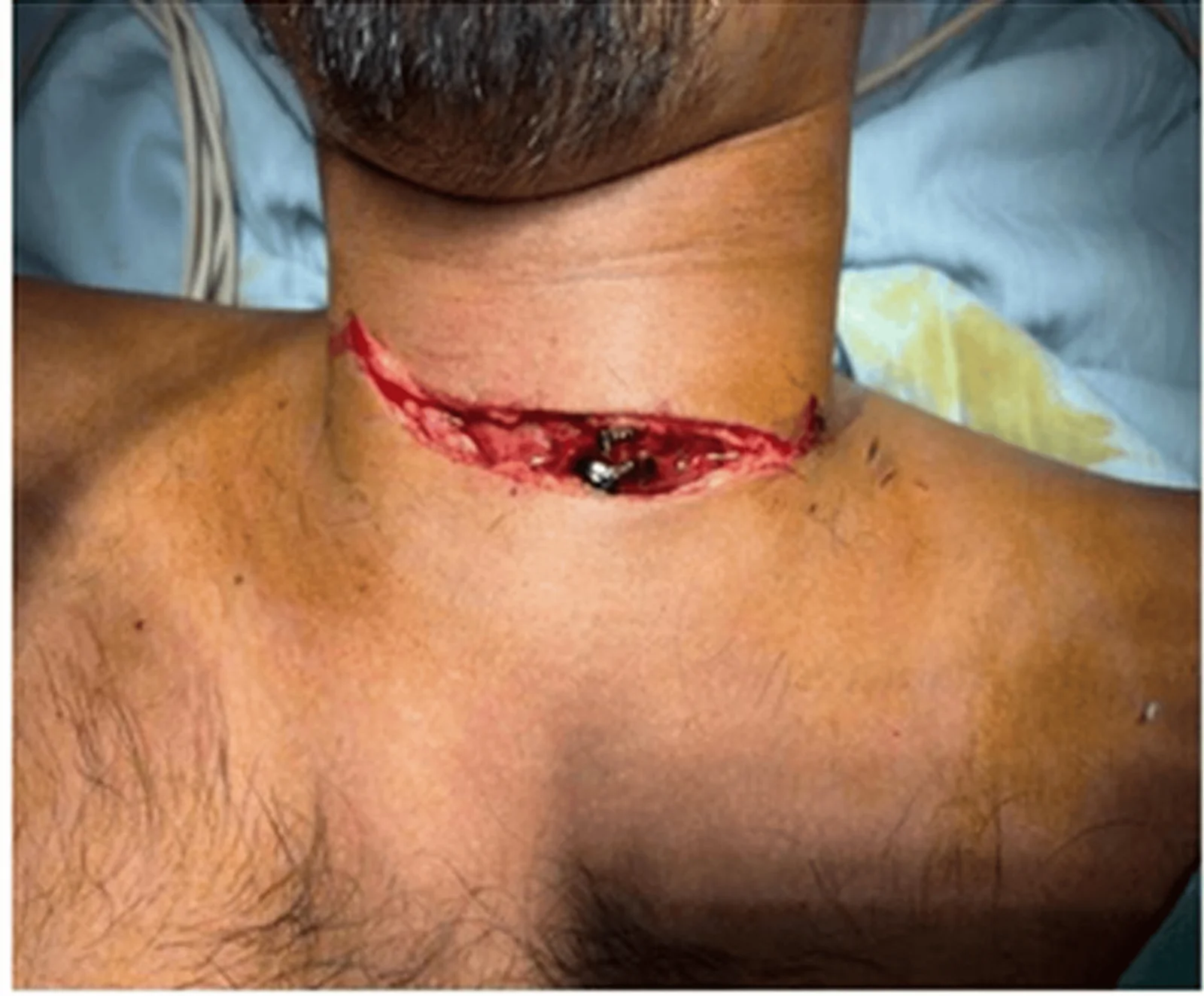
Clinical photograph showing a horizontal cut-throat injury over the thyroid cartilage following a road traffic accident involving a fall onto roadside fencing wire. The wound measured approximately 12 × 3.5 cm and was associated with a C3 vertebral body fracture.

The placement beyond the injured segment was confirmed from the following points: 1) auscultation on both sides of the chest was confirmed with bilateral equal air entry; 2) no leakage of air through the injured segment during positive pressure ventilation was confirmed; 3) confirmation of the endotracheal tube in the trachea was confirmed by the surgeons, ensuring that the tube had not reached any false tract, especially during the time when the tube was being placed distally to the injured segment.

Ventilation used low tidal volumes and minimal positive end-expiratory pressure (PEEP). An arterial catheter was inserted. The ENT team performed tracheostomy and repaired the tracheal injury. The patient was transferred to intensive care with the tracheostomy in situ and had a stable immediate postoperative course. There were no hemodynamic, airway or ventilation-related perioperative compromises to the patient and the surgery was uneventful.

Case 2

A 25-year-old man presented several hours after a self-inflicted razor-blade neck wound. The approximately 12 × 4 cm infrahyoid wound was above the true vocal cords; the vocal cords and divided thyroid cartilage were visible (Figure 2). He was alert and restless, with pulse 110/min, blood pressure 110/70 mmHg and SpO₂ 95%. Oxygen, two intravenous lines, fluids and blood preparation were provided. After preoxygenation, anaesthesia was induced with fentanyl, propofol and suxamethonium. Propofol and suxamethonium were administered to facilitate rapid tracheal intubation under direct laryngoscopic vision. Suxamethonium was selected for its rapid onset and relatively short duration of action. He underwent preoxygenation before induction. Direct laryngoscopy permitted careful advancement of a smaller-diameter tracheal tube beyond the injured airway segment. Sterile gauze was placed over the external wound to minimise air leakage. Emergency tracheostomy was subsequently performed in both patients, followed by definitive surgical repair. A 7.0-mm tracheal tube was advanced beyond the injured segment under direct vision. Tracheostomy was then performed and an 8-mm tube inserted while the tracheal, laryngeal and soft-tissue injuries were repaired (Figure 3). There were no hemodynamic, airway or ventilation-related perioperative compromises to the patient and the surgery was uneventful. He remained ventilated for approximately 12 hours. The tracheostomy was removed on postoperative day 10 and he was discharged on day 16 with normal phonation, swallowing and breathing.

**Figure 2:**
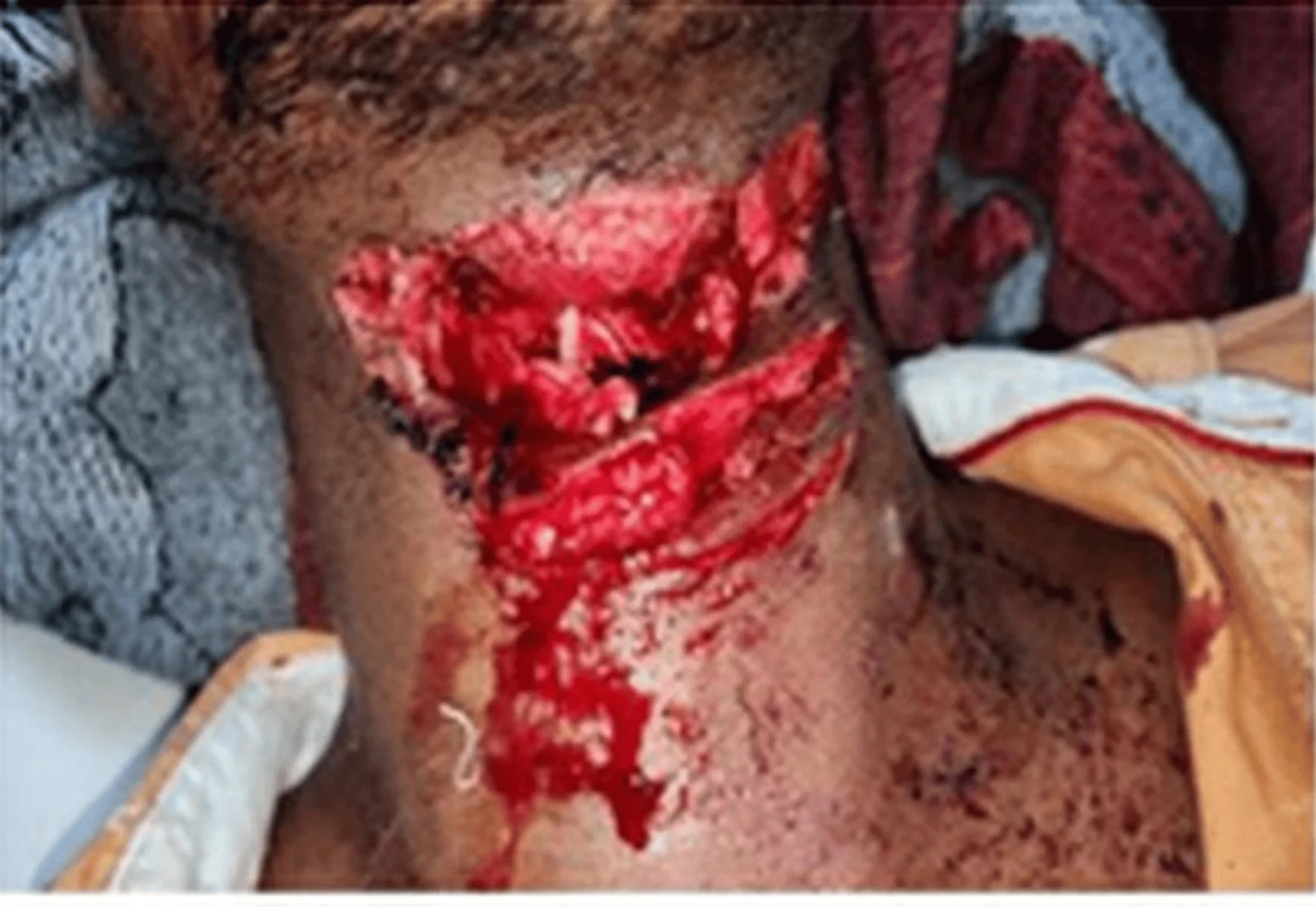
Clinical photograph showing an approximately 12 × 4 cm self-inflicted infrahyoid neck wound following a razor-blade injury. The wound was situated above the true vocal cords, with the divided thyroid cartilage and vocal cords visible.

**Figure 3:**
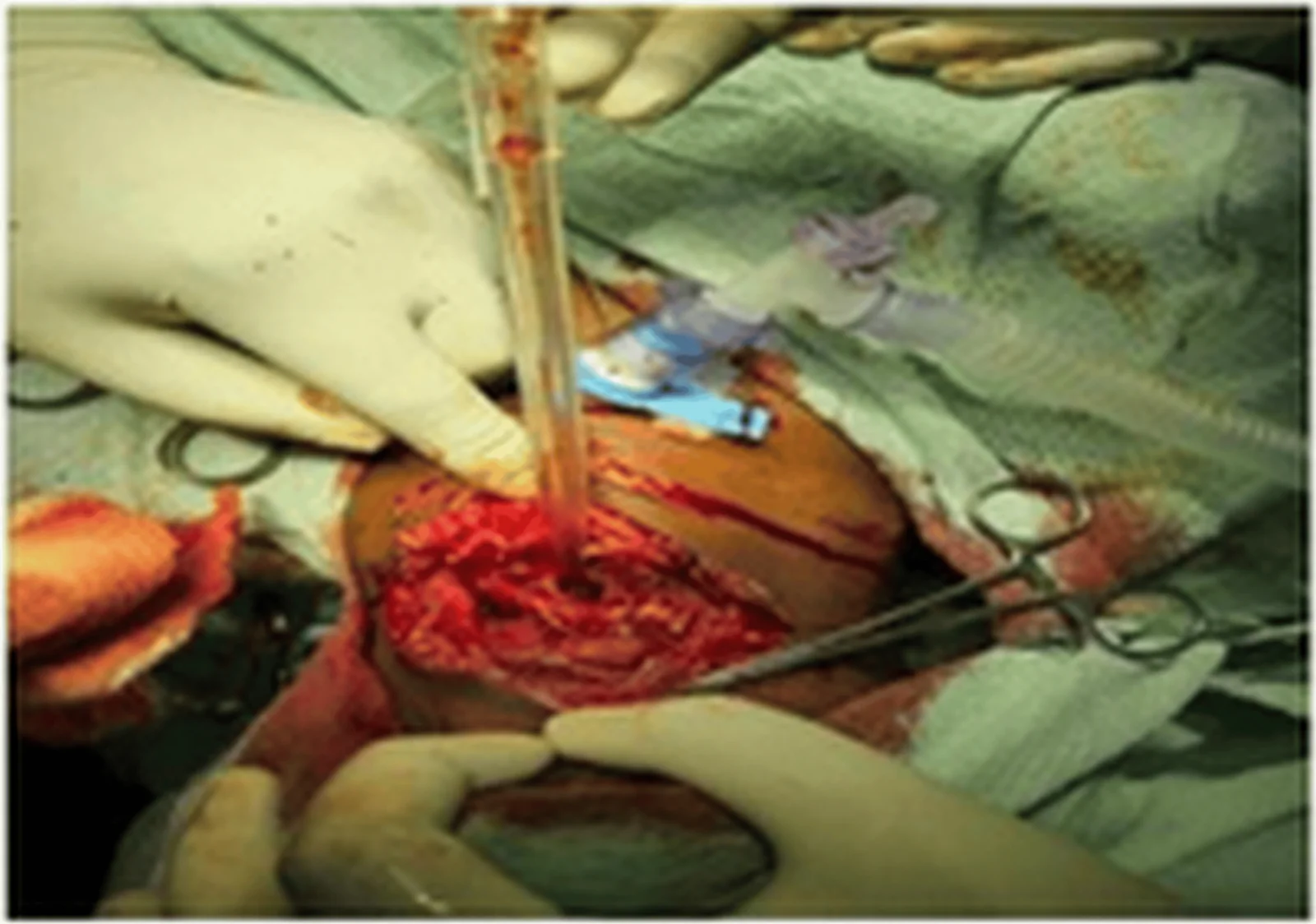
Intraoperative photograph showing surgical exploration and repair of the penetrating anterior neck injury in Patient 2 following airway control and tracheostomy.

Case 3

A 20-year-old man with severe depression presented after a self-inflicted knife wound measuring approximately 7 × 3 cm (Figure 4). He was agitated and in pain, with dyspnoea and difficulty phonating; pulse was 154/min, blood pressure 90/60 mmHg and room-air SpO₂ 90%. Oxygen, two intravenous cannulas, crystalloid therapy and preparation of two units of blood were undertaken before emergency exploration. After preoxygenation, anaesthesia was induced with fentanyl, propofol and suxamethonium. Direct laryngoscopy permitted careful advancement of a 7.0-mm tracheal tube beyond the injured segment. Emergency tracheostomy was subsequently performed. Exploration showed the thyroid cartilage divided into three fragments above the true vocal cords, with laryngeal mucosal and soft-tissue injury. Bleeding vessels were coagulated and the injured tissues were repaired. A nasogastric tube was inserted. There were no hemodynamic, airway or ventilation-related perioperative compromises to the patient and the surgery was uneventful. The patient was ventilated for 18 hours, then weaned. Enteral feeding was started within 12 hours and psychiatric assessment was obtained on postoperative day 5. The tracheostomy was removed on day 6 and he was discharged on day 8 with normal speech.

**Figure 4:**
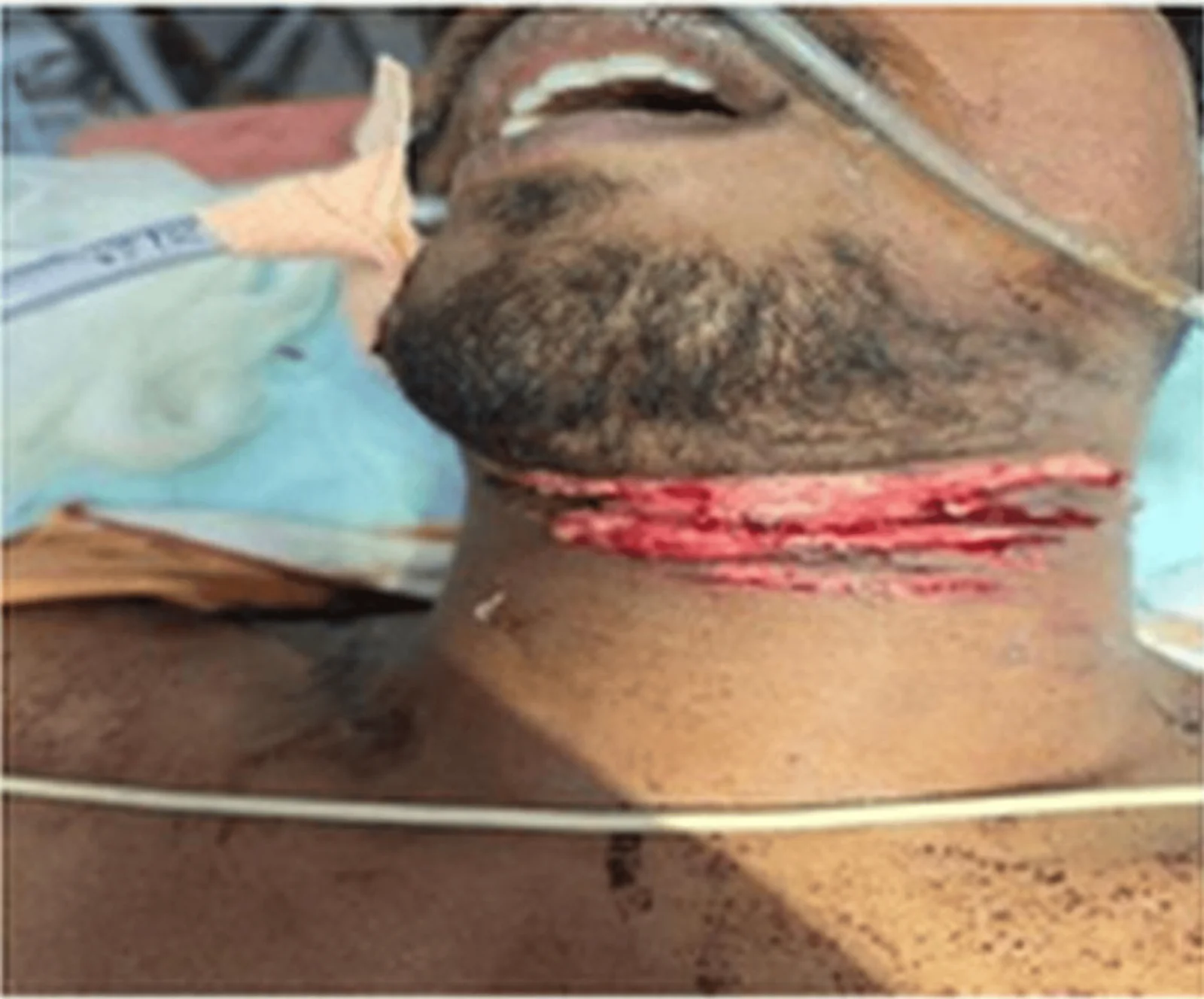
Clinical photograph showing an approximately 7 × 3 cm self-inflicted neck wound following a knife injury. Surgical exploration subsequently demonstrated fragmentation of the thyroid cartilage with associated laryngeal mucosal and soft-tissue injury.

Surgical and functional outcomes

All three underwent tracheostomy and repair without documented intraoperative airway complications. Patients 2 and 3 were decannulated and discharged with preserved function; patient 1 remained stable postoperatively.

**Table 1:** Clinical and airway management characteristics of the three patients

Characteristic	Case 1	Case 2	Case 3
Mechanism of injury	Road traffic accident/fencing wire	Self-inflicted razor blade	Self-inflicted knife
Major associated injury	C3 vertebral fracture; thyroid cartilage laceration	Divided thyroid cartilage; vocal cord involvement	Thyroid cartilage fragmentation; laryngeal mucosal injury
Initial SpO₂	85% (room air)	0.95	90% (room air)
Airway technique	Video laryngoscopy planned with manual in-line stabilisation; tracheal tube advanced beyond the injury	Direct laryngoscopy; tracheal tube advanced beyond the injury	Direct laryngoscopy; tracheal tube advanced beyond the injury
Tracheal tube	7.5 mm	7.0 mm; subsequently 8.0 mm tracheostomy tube	7.0 mm
Definitive airway	Tracheostomy	Tracheostomy	Tracheostomy
Postoperative ventilation	Yes; duration not stated	Approximately 12 hours	18 hours
Outcome	Stable immediate postoperative course	Discharged on day 16; normal phonation, swallowing, and breathing	Discharged on day 8; normal speech

## Discussion

These cases illustrate that airway management in penetrating neck trauma must be individualised according to the level of airway disruption, associated cervical or laryngeal injury and physiological status. Contemporary reviews emphasise rapid assessment for hard signs of aerodigestive or vascular injury and multidisciplinary planning, while avoiding unnecessary manipulation of a potentially unstable airway [1,2].

In the first patient, the associated C3 vertebral fracture required limitation of cervical movement. Video laryngoscopy with manual in-line stabilisation was planned, with a surgical airway immediately available. In the other two patients, direct laryngoscopy permitted careful advancement of a smaller tracheal tube beyond the injured segment (Table 2). Previous reports similarly describe airway control with direct or video laryngoscopy while the surgical team assists with passage of the tube beyond the site of tracheal disruption [4,5]. Controlled advancement under direct vision is important because a tracheal tube can enter a false passage through a tracheal tear [3].

**Table 2:** Table showing patients with different laryngoscopic modes and subsequent outcome

Clinical characteristic / outcome	Number of patients (n=3)	Percentage (%)
Video laryngoscopy done	1/3	33.3%
Direct laryngoscopy used	2/3	66.7%
Tracheal tube advanced beyond the injured segment	3/3	100.0%
Definitive tracheostomy performed	3/3	100.0%
Postoperative mechanical ventilation required	2/3	66.7%
Satisfactory airway/functional outcome	3/3	100.0%

An important consideration in these injuries is that airway management should be viewed as a planned sequence rather than as a single intubation attempt. Plan A should consist of the least traumatic technique that is likely to provide access to the distal intact airway, based on the anatomical level of disruption, cervical spine status and physiological condition. In our series, this consisted of video laryngoscopy with manual in-line stabilisation in the patient with cervical spine injury and direct laryngoscopy with careful distal tube advancement in the other two patients. Immediate surgical assistance should be considered an integral component of Plan A rather than a measure reserved only for failure. The anticipated complications include worsening of the airway disruption, creation of a false passage, increased air leakage, loss of ventilation and further tissue trauma during instrumentation.

Plan B should therefore be prepared before induction and should involve immediate surgeon-assisted airway control if the tube cannot be advanced safely beyond the disrupted segment or if effective ventilation cannot be established. Depending on the level and extent of injury, this may require direct surgical exposure of the trachea and emergency tracheostomy. If both oral/nasal intubation and the planned surgical approach fail to provide oxygenation, Plan C should consist of an appropriate emergency front-of-neck rescue strategy determined by the anatomy of the injury and the available surgical expertise. This contingency-based approach is particularly important because repeated blind attempts at intubation may worsen disruption or create a false passage.

Definitive tracheostomy was performed in all three patients, providing a secure airway while allowing repair of the laryngotracheal injuries. Low tidal volumes and minimal PEEP were used in the first patient to limit excessive airway pressure and air leakage. Continuous oxygen saturation, capnography and haemodynamic monitoring were maintained, with invasive arterial monitoring in the patient with associated cervical injury. Postoperative intensive care allowed controlled weaning and surveillance for respiratory complications.

Point-of-care ultrasonography (POCUS) may provide additional real-time information regarding the position of the trachea, tracheal rings and cricothyroid membrane and may assist planning of tracheostomy or emergency front-of-neck access when external anatomical landmarks are distorted. Fibre-optic transillumination can provide complementary information regarding the course of the trachea. Arora et al. described the combined use of POCUS and transillumination to delineate tracheal deviation during challenging tracheostomy, demonstrating how these techniques may assist surgical airway localisation in anatomically distorted necks [9].

Although immediate airway control takes priority in an unstable or hypoxaemic patient, selected anatomically challenging cases may benefit from adjunctive techniques when sufficient time and physiological stability permit. Three-dimensional reconstruction of pre-existing CT imaging with virtual endoscopy can provide a spatial representation of complex airway anatomy and may assist in anticipating the course of a distorted or narrowed airway. Virtual endoscopy using OsiriX (Pixmeo SARL, Bernex, Switzerland) has been described as a non-invasive method of pre-emptive airway mapping in patients with complex head and neck pathology [10]. Such imaging should be regarded as a planning adjunct rather than a prerequisite for emergency airway control.

A broader review of POCUS in airway management has described its utility for airway assessment and identification of relevant anatomical structures [11]. These modalities should not delay definitive airway control in patients with severe hypoxaemia, rapidly progressive airway compromise or ongoing major bleeding.

Nutritional support and psychological assessment were also incorporated into postoperative care when indicated. Rehabilitation following successful airway repair is an important component of overall recovery and should extend beyond securing the airway. Follow-up should assess airway patency, respiratory function, swallowing, phonation and readiness for decannulation. In our series, Patients 2 and 3 were successfully decannulated and discharged with preserved swallowing, breathing and speech/phonation. Patient 3 also underwent psychiatric assessment, which was particularly relevant given the self-inflicted mechanism of injury. These aspects emphasise that successful management of penetrating laryngotracheal trauma includes restoration of functional airway, swallowing and communication outcomes in addition to anatomical repair.

The principal limitation of this report is the small number of patients and the heterogeneity of the injuries; the cases therefore cannot establish the superiority of one airway technique. Their value lies in demonstrating that the airway plan should be anatomy and physiology-driven, with immediate access to a surgical airway and close communication between anaesthesia and ENT/trauma teams. Recent case literature continues to emphasise this individualised approach in penetrating tracheal injury [3-5]. The practical lesson from these cases is therefore not that one intubation technique is universally preferable, but that successful management depends on anticipating failure, securing access to the distal airway with minimal manipulation, maintaining a clearly defined surgical rescue pathway and subsequently addressing functional recovery.

## Conclusions

In this three-patient case series of penetrating anterior neck injuries with laryngotracheal disruption, successful airway management was achieved by adapting the intubation strategy to the individual injury pattern and physiological status. Video laryngoscopy with manual in-line stabilisation was performed in the patient with an associated C3 vertebral fracture, while direct laryngoscopy with careful advancement of a smaller tracheal tube beyond the injured segment was used in the other two patients. Definitive tracheostomy and surgical repair were subsequently performed in all three patients. Two patients required short-term postoperative mechanical ventilation, and all three achieved satisfactory airway and functional outcomes. These cases highlight the importance of controlled distal airway access, avoidance of forceful manipulation across the disrupted segment, immediate availability of surgical airway rescue and close coordination between anaesthesia and ENT teams. In conclusion, penetrating anterior neck injuries with laryngotracheal disruption require rapid but controlled airway management. Careful distal airway control, immediate readiness for surgical airway access, definitive repair and vigilant postoperative care supported favourable outcomes in these three patients.
